# Clinical tips to optimize liquid nitrogen spray cryotherapy in esophageal malignancy

**DOI:** 10.1093/dote/doag024

**Published:** 2026-03-17

**Authors:** Joseph Spataro, Michael Fuchs, Alvin Zfass

**Affiliations:** Department of Internal Medicine, Division of Gastroenterology, Richmond Veterans Affairs Medical Center, Central Virginia VA Health Care System, Richmond, VA, USA; Department of Internal Medicine, Division of Gastroenterology, Hepatology, and Nutrition, Virginia Commonwealth University Health System, Richmond, VA, USA; Department of Internal Medicine, Division of Gastroenterology, Richmond Veterans Affairs Medical Center, Central Virginia VA Health Care System, Richmond, VA, USA; Department of Internal Medicine, Division of Gastroenterology, Hepatology, and Nutrition, Virginia Commonwealth University Health System, Richmond, VA, USA; Department of Internal Medicine, Division of Gastroenterology, Richmond Veterans Affairs Medical Center, Central Virginia VA Health Care System, Richmond, VA, USA; Department of Internal Medicine, Division of Gastroenterology, Hepatology, and Nutrition, Virginia Commonwealth University Health System, Richmond, VA, USA

**Keywords:** cryotherapy, esophageal neoplasms

## Abstract

Liquid nitrogen spray cryotherapy is an approved modality that has been used to treat esophageal disease for more than 20 years. This versatile tool effectively ablates unwanted tissue; however, its widespread adoption has been limited by procedural complexity limiting technical success. Drawing on the collective experience of the authors, this article provides practical tips to optimize procedure safety, efficiency, and outcomes, with a focus on esophageal malignancy.

## INTRODUCTION

Nitrogen freezes at −196°C and expands rapidly from liquid to gas (1:694). This unique property can be harnessed to destroy cancer! Liquid nitrogen spray cryotherapy (LNSC) is primarily used in the management of dysplastic Barrett’s esophagus and to palliate esophageal malignancy. Emerging applications include benign esophageal strictures, vascular disorders of the stomach and rectum, such as gastric antral vascular ectasia and radiation proctopathy.

The authors have collectively performed over 500 LNSC procedures. This manuscript focuses on LNSC in esophageal malignancy. The aim is to share procedural tips to help clinicians optimize technique and improve outcomes. This article supplements the standard instructions and operator manuals with insights gained from clinical experience. These suggestions represent the authors’ experience and should be interpreted based on the reader’s preferences and do not represent a universal approach.

## PRE-PROCEDURE

### The patient

Effective preparation begins with patient and family education. A detailed informed consent should cover the specific risks and benefits. There is the need to establish an early alliance with their spokesperson/family. They will be involved in decision-making and the primary contact immediately after the procedure. Managing expectations will be important since the outcomes may not be immediate. The patient and their trusted individual(s) should understand that therapeutic effects may take several days to manifest as tissue effects occur.

### Equipment

The nitrogen tank should be completely filled. Experience and physics (Boyle’s Law) dictate that unfilled tanks may present with low pressure.

If the lumen of the esophagus allows, attach a proper-fitting clear distal cap to the endoscope to improve stability and visualization. Ensure the side hole is covered by properly positioning the cap on the endoscope to prevent fluid egress onto the lens. In a narrowed lumen esophagus, smaller caliber endoscopes may be needed. The 2.1 mm spray catheter will fit the channel of most gastroscopes and neonatal endoscopes. Endoscope/cap compatibility will differ. Check manufacturer manual for size and capability as fittings may differ.

### Medication

Administration of glycopyrrolate, an anti-cholinergic, reduces secretions to help maintain a clear field.

## PROCEDURAL TECHNIQUES

### Angle of spray (i.e. upstream or downstream)

The spray catheter may be positioned proximal or distal to the area of interest. Either is acceptable providing that a uniform ‘frost’ (i.e. snow-with appearance of the freeze) can be accomplished.

### Spray duration and intervals

The issue of spray time is a matter of controversy. All recommendations related to dosimetry are based on individual preference. There is no scientific evidence which dictates the exact spray time. Further studies are required to establish proper timing and dose–response curves (i.e. dosimetry).

It is our practice when treating malignancy to schedule three consecutive sessions of LNSC at 1–2-week intervals. Response is assessed. Since this is palliative, patient preference dictates future management. A realistic endpoint is the ability to swallow liquids and not necessarily solids. If sustained improvement, future management incorporates patient preference, treatment efficacy, and the risks and benefits.

## TROUBLESHOOTING DURING THE PROCEDURE

### Patient monitoring

Venting is critical. The rapidly expanding nitrogen gas carries risks, therefore, requires continuous active venting (i.e. suction) through the CryoDecompression Tube (CDT). Suction should be continued through the cryospray and defrost cycles. CDT positioning is critical and should be confirmed before starting cryotherapy.

The endoscopist should be vigilant and recognize the problem of expanding gas. This is accomplished by an assistant or two keeping a hand on the abdomen to detect distention and the other on the neck for crepitus. Detection of these events requires immediate cessation of cryospray.

### Proper CryoDecompression tube placement

The standard recommendation is to position the CDT using the black bands within the cardioesophageal junction. Once positioned, our preference is to insert the venting tube 2–3 centimeters beyond into the stomach to ensure decompression. Either manual fixation, marking, or wrapping the CDT with tape at the mouth documents position.

### Avoiding mucosal adherence

Increased suction pressures may indicate mucosal adherence of the CDT. This condition may be suggested by pressures exceeding 13 inHg on the console monitor or by the presence of an audible cue. In our experience, a 1–2 cm back-and-forth motion combined with gentle rotation of CDT during nitrogen delivery prevents mucosal adherence and facilitates decompression.

The frozen catheter or endoscope ‘sticking-to-the-mucosa’ is a troublesome phenomenon like ‘a warm tongue on a frozen pole’. To avoid this, create a ‘frost,’ as in the ‘ski-slope’ analogy, which decreases this friction and ‘stickiness’ allowing freedom of motion. Therefore, before freezing the target, spray the proximal surface (to avoid adherence). This minimal surface frost will act as a lubricant to allow motion of the endoscope and will not injure the normal mucosa. Additionally, keeping the spray catheter inside the distal attachment cap avoids inadvertent catheter/mucosal contact.

Contact/adhesion may cause bleeding and obscure vision. Stop spraying, and do not dislodge the catheter! Wait until the catheter thaws (without using a defrost cycle) before continuing. This may take 10–15 seconds.

### Cannot freeze and cannot see

Ice accumulation within the working channel of the endoscope or on the lens interferes with gas delivery and obscures vision. Maintaining a relatively dry channel and clear lens is essential for success.

Prepare the endoscope before spraying. Before intubation, clear excess moisture from the working channel by flushing with isopropyl alcohol followed by air to ensure a clean, dry channel. Once evaporated, alcohol no longer poses a caustic risk. Wipe the lens with isopropyl alcohol to reduce the risk of frost formation. After endoscope insertion, use a defrost cycle (‘warmed’ nitrogen gas) with active venting while advancing the spray catheter through the working channel.

To maintain channel patency and optics, do not suction. Avoid irrigation or lens rinses during the spray. Consider insufflation with carbon dioxide during the delivery of nitrogen which may minimize frost on the lens. Vision may not be optimal, but with experience, absolute vision is not absolutely necessary!

Fluid in the esophagus may freeze, hindering visualization and treatment. Position the patient’s head upright to allow gravity-assisted drainage. Short duration sprays from the proximal (oral) side toward the distal end helps move fluid downstream and prevents its return to the lens.

Acetic acid (2%–3%), which enhances mucosal detail, is also used for its mucolytic action to enhance treatment efficacy.

In the narrowed esophagus, the venting tube may obscure visualization. In such cases, gently retract the endoscope to a more proximal location and use the tip of the endoscope to position the tube and improve visibility.

Spray failure, or ‘spray dropout,’ may occur (i.e. no flow of nitrogen). This is recognized by the absence of mucosal frost that is usually accompanied with a change in the sound/optics of spray delivery. If an ice buildup on the spray catheter has occurred, use the defrost cycle to thaw the catheter and to restore free movement within the channel. If spray delivery remains impaired due to the ice buildup, the spray catheter should be withdrawn and the endoscope working channel dried. The catheter is then reinserted and an additional defrost cycle is performed. The spray catheter may need to be replaced if spray failure persists.

In addition to the endoscope, the console may present issues related to low temperatures. Spray dropout may occur when console internal temperatures fall below −120°C and fluid within the endoscope freezes. This is physics! The console operator should carefully monitor system temperatures to ensure that they remain above this critical level. This is accomplished by performing preventative defrost cycles between sprays.

### Difficulty with treating the Cardioesophageal junction/gastric cardia

Treating the cardioesophageal junction is challenging because it requires using a forward-firing spray catheter with nearly 180^°^ tip deflection…in a frozen endoscope. Flexion and maneuverability are limited!

Before intubation, attach a clear distal cap to the endoscope. In addition, confirm that the endoscope can be retroflexed in approximately 180^°^ with the catheter in place (i.e. at end of working channel or distal cap).

Cardia tumors can be treated with a straight or retroflexed endoscope. The authors’ preference is to use retroflexion first. After inserting the catheter-loaded endoscope into the stomach, practice retroflexion to identify the optimal position for freezing. Wait several seconds after spraying to allow for cardiofundal distention to improve visualization.

During the spray, keep the spray catheter tip within the cap to enhance precise spray delivery and prevent mucosal adherence. In selected cases, lifting agents on the target may improve access. If esophageal contractions interfere, intravenous glucagon (0.5 mg–1 mg) relaxes smooth muscle and restores stable visualization.

## SPECIFIC ANATOMY

### Upper Esophageal malignancy

Malignancy in the upper esophagus requires special attention.

Due to the proximity of the esophagus to the airway, nitrogen can displace oxygen and may cause sudden desaturation. If this occurs, spraying should be stopped to allow recovery. When resuming, use short spray intervals of 10 seconds, gradually increasing to 20 seconds over two to three cycles if tolerated.

The upper esophagus is also at higher risk for complications because of its thin muscularis propria. Pre-cryotherapy dilation may be needed to allow passage of the CDT. Caution should be used when addressing the anterior wall due to the risk of tracheoesophageal fistula.

Key endoscopic landmarks (i.e. vocal cords and left mainstem bronchus) help orient the anterior wall of the esophagus. Tattoo marking may be considered to guide current and future treatments.

### Patients with gastrostomy tube

Spray cryotherapy can be safely performed routinely in patients with a gastrostomy tube after the tract has matured (typically four weeks). Venting through a functioning gastrostomy tube aids decompression and reduces abdominal distention. If the esophageal lumen is severely narrowed, the venting tube can be introduced via a gastrostomy tube if size allows. Alternatively, the gastrostomy tube may be temporarily removed and the CDT placed through the stoma directed with the tip angled toward the fundus.

### Top pearls

- Stay attentive to procedural sounds (i.e. ambient noise) and consistently monitor both patient status and console data.
- Closely observe the patient’s abdomen for distention and the neck for crepitus, as these signs may indicate gas-related complications.
- Prevent the spray catheter adhering to the mucosa. Position the tip of the cryospray catheter within the protective distal attachment cap space to avoid adherence. If adherence occurs, keep the endoscope and catheter still, STOP airflow, allow thawing, then gently remove the catheter to avoid tissue injury.
- If technical malfunctions arise that cannot be resolved, abort the procedure.

### Personal comment

Patient selection at our Veterans Affairs facility is unique. The authors have primarily used spray cryotherapy to treat cancer. The outcomes are often challenging. One must prepare to confront the natural progression of terminal disease while embracing the role to bring hope and comfort. It is indeed a privilege to practice the majesty of medicine to promote healing and hope.

## Notes

Specific author contributions: J.S.: Drafted the article, critically reviewed. M.F.: Critically reviewed the article. A.Z.: Drafted the article, critically reviewed. All authors reviewed and approved the final article.

Financial support: No funding was received for the manuscript.

